# Peripheral myelin protein 2 – a novel cluster of mutations causing Charcot-Marie-Tooth neuropathy

**DOI:** 10.1186/s13023-019-1162-x

**Published:** 2019-08-14

**Authors:** Paulius Palaima, Teodora Chamova, Sebastian Jander, Vanyo Mitev, Christine Van Broeckhoven, Ivailo Tournev, Kristien Peeters, Albena Jordanova

**Affiliations:** 1 Molecular Neurogenomics group, VIB-UAntwerp Centre for Molecular Neurology, University of Antwerp, Antwerp, Belgium; 2 Department of Neurology, Medical University-Sofia, Sofia, Bulgaria; 3 Department of Neurology, Heinrich Heine University Duesseldorf, Medical Faculty, Duesseldorf, Germany; 4 Department of Medical Chemistry and Biochemistry, Medical University-Sofia, Sofia, Bulgaria; 5 Neurodegenerative Brain Diseases group, VIB-UAntwerp Centre for Molecular Neurology, University of Antwerp, Antwerp, Belgium; 6 Laboratory of Neurogenetics, Institute Born-Bunge, University of Antwerp, Antwerp, Belgium; 7 Department of Cognitive Science and Psychology, New Bulgarian University, Sofia, Bulgaria

**Keywords:** Demyelinating, CMT, PMP2, Novel, Cluster

## Abstract

**Background:**

Charcot-Marie-Tooth (CMT) disease is the most common inherited neuromuscular disorder characterized by wide clinical, genetic and pathomechanistic heterogeneity. Recently, the gene encoding peripheral myelin protein 2 (*PMP2)* was identified as a novel cause for CMT neuropathy with three mutations that structurally cluster together (p.Ile43Asn, p.Thr51Pro, p.Ile52Thr) reported in five families.

**Results:**

Using whole exome sequencing and cohort screening we identified two novel missense substitutions in *PMP2* in Bulgarian (p.Met114Thr, c.341C > T) and German (p.Val115Ala, c.344 T > C) families. The mutations affect adjacent and highly conserved amino acid residues outside of the known mutation-rich region in the protein. Crystal structure analysis positions the affected residues within a cluster of highly conserved fatty acid coordinating residues implying their functional significance. The clinical, electrophysiological and imaging features in both families were consistent with a childhood onset polyneuropathy with variable patterns of demyelination, slow to very slow progression, and most severe involvement of the peroneal muscles.

**Conclusions:**

We expand the genetic and phenotypic spectrum of *PMP2*-related peripheral neuropathy. Our findings reveal a second mutational cluster in the protein.

**Electronic supplementary material:**

The online version of this article (10.1186/s13023-019-1162-x) contains supplementary material, which is available to authorized users.

## Background

Charcot-Marie-Tooth disease (CMT) represents a genetically and phenotypically highly heterogeneous group of disorders of the peripheral nervous system, affecting 9.7–82.3/100,000 individuals in the European population [1]. All forms of inheritance are observed in CMT and mutations in over 80 genes with diverse functions have been described as disease-causing [2].

Based on histology and electrophysiology three main types of CMT are recognized: demyelinating (CMT1), axonal (CMT2) and intermediate (I-CMT). CMT1 is characterized by loss of myelin and nerve conduction velocities (NCVs) below 38 m/s in the median motor nerve. CMT2 primarily affects the axons with patients showing normal or slightly reduced NCVs (> 38 m/s) [3, 4]. Finally, individuals showing signs of both demyelination and axonal degeneration, with NCVs between 25 and 45 m/s, are classified under I-CMT [5].

CMT1 accounts for 40–50% of all CMT patients [2]. The disease affects the myelin sheath, a complex structure made out of layers of highly compacted Schwann cell membrane. It is very sensitive to changes in its protein or lipid composition and alterations lead to inefficient compaction and insulation of the axons resulting in a significant reduction in NCVs [6]. Over 50% of the total peripheral myelin is made up of four proteins: myelin basic protein (MBP), myelin protein zero (MPZ), peripheral myelin protein 2 (PMP2) and peripheral myelin protein 22 (PMP22) [7]. A genomic duplication that includes *PMP22* was the first identified genetic cause of CMT (CMT1A) [8, 9]. Overall, mutations affecting PMP22 and MPZ account for 76% of all CMT1 patients [10]. While *MBP* has not been implicated in CMT so far, *PMP2* has recently emerged as a novel rare cause of dominant CMT1 [11–14].

**Fig. 1:**
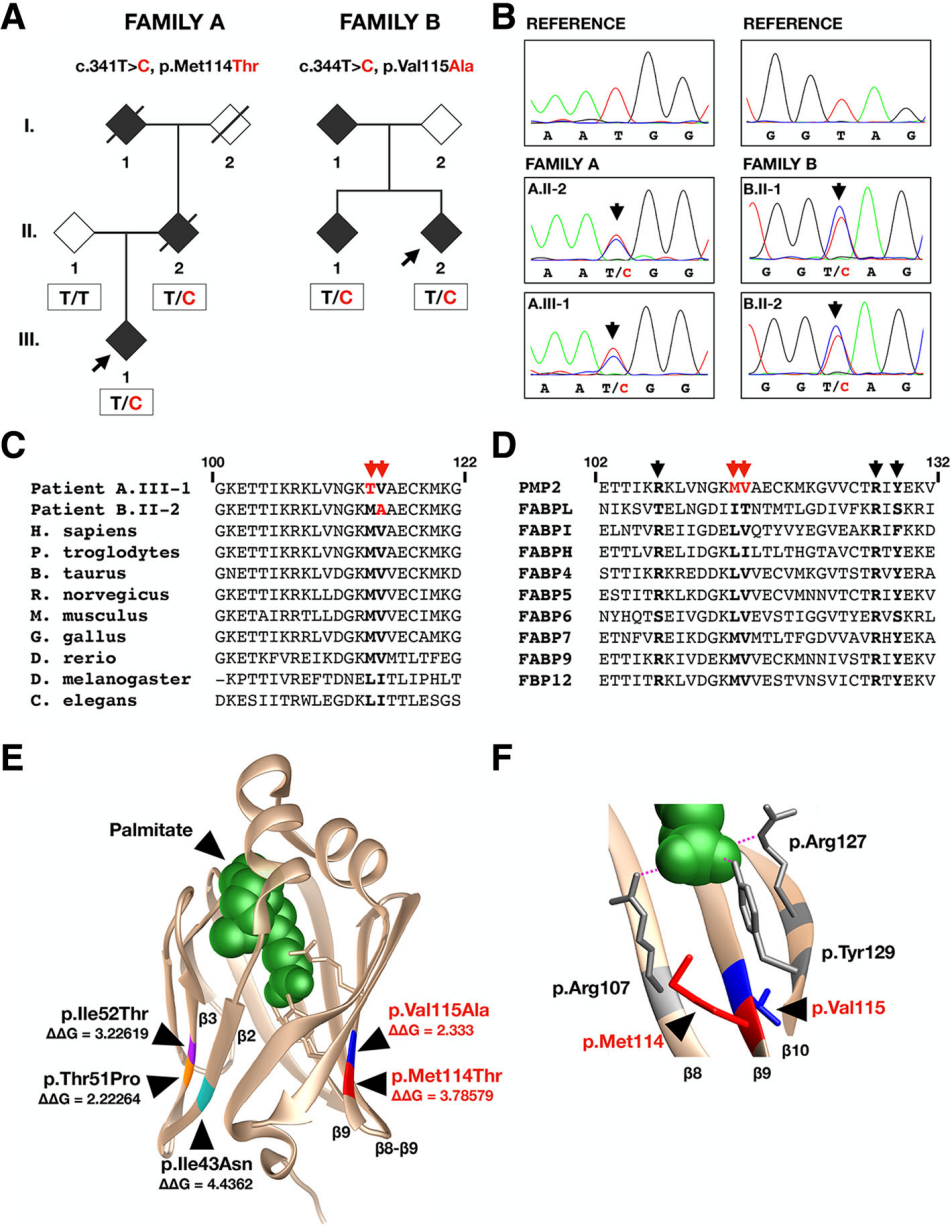
**a** Pedigrees and segregation analysis of the Bulgarian (Family A) and German (Family B) families. The mutated residue is indicated in red in the available genotyped individuals. Black diamonds indicate clinically affected individuals. Black arrows indicate probands. **b** Electropherograms of c.341 T > C (Family A) and c.344 T > C (Family B) changes in the two families carrying novel *PMP2* mutations. **c** The evolutionary conservation of the amino acids affected by the newly identified mutations (red arrows) in PMP2. **d** The location of the two affected residues (red arrows) in relation to the fatty acid coordinating residues (black arrows). **e** Position of the two mutational clusters on the crystal structure of PMP2. The novel mutations are indicated in red (p.Met114Thr) and blue (p.Val115Ala) on the protein structure. “ΔΔG” values are provided for each known CMT-causing mutation (FoldX). **f** Fatty acid coordinating residues (p.Arg107, p.Arg127, p.Tyr129) surrounding the two mutated amino acids on the crystal structure of PMP2. Palmitate is indicated in green

PMP2 is a small 14 kDa protein belonging to the fatty acid binding protein family (FABP) [15]. It is involved in remyelination, stiffening of the myelin sheath and has a suggested role in membrane stacking and lipid transfer [15–18]. Recently, three heterozygous missense mutations in *PMP2* were identified in five families with dominant CMT1 of European (p.Ile43Asn, c.128 T > A; p.Thr51Pro, c.151A > C; p.Ile52Thr, c.155 T > C) and Asian (p.Ile43Asn, c.128 T > A) descent [11–14]. Notably, all mutations identified so far cluster in adjacent positions on two neighbouring beta-strands of the PMP2 crystal structure and were observed to affect the functional dynamics of the protein (Fig. 1e) [13, 16].

Here, we describe the identification of two novel disease-causing mutations in *PMP2* in a Bulgarian and a German family.

## Results

### Clinical representation

#### Family A

**Fig. 2:**
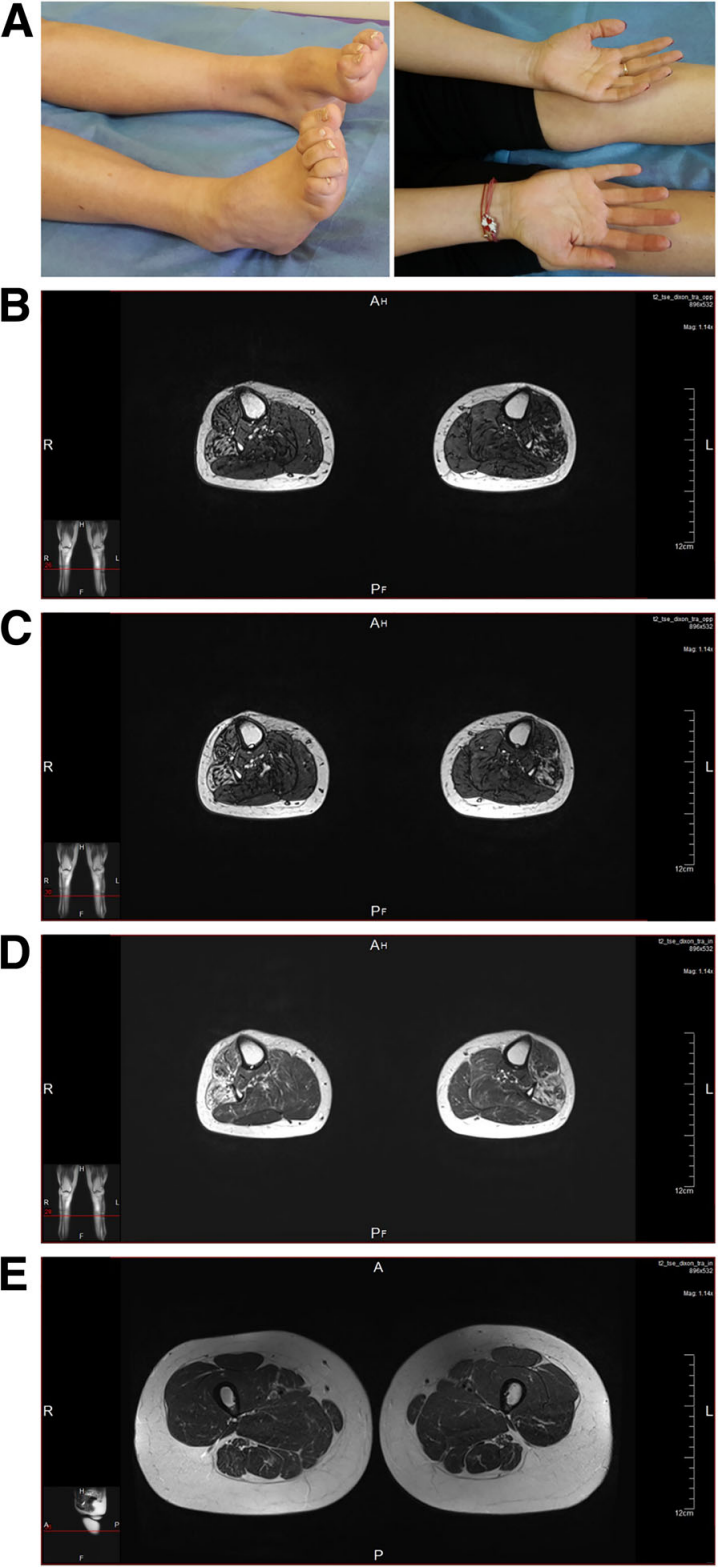
**a** Clinical features of individual A.III-1, aged 37 years, demonstrating bilateral *pes cavus*, distal hypotrophies in both calves and muscle hypotrophies in the hands, more pronounced for the thenar muscle in comparison with the hypothenar ones. **b**-**e**. Magnetic resonance images of individual A.III-1 performed at the age of 37 years. Non-enhanced T1, T2 and proton density (PD) images are shown. **b**-**d**. The lateral compartments of the calves, including peroneus longus and brevis muscles are more severely affected, followed by *extensor digitorum brevis* and *tibialis anterior* muscles. The posterior compartment of the calves seems relatively preserved. **e** At thigh level the muscles are preserved as well, with slight changes visible in semimembranosus and semitendinosus muscles

The proband is a 37-year old Bulgarian female (A.III-1) reporting a family history of CMT consistent with autosomal dominant inheritance, with both an affected parent and grandparent (Figs. 1a, 2a). The disease onset of patient A.III-1 was in childhood, with delayed walking at the age of 18 months with frequent falls (Table 1). She had always run slower compared to her peers. She has had difficulties in stepping on her heels and toes since the age of 2–3 years. Foot deformities (high arched feet, thin calves) became apparent in the first decade of life. During her teens proximal muscle weakness in the lower limbs was noticed with difficulties in climbing stairs. The patient was referred to the department of neurology at the age of 20 and was followed up for 17 years. From the age of 29 years she started having weakness in the distal hand muscles with impairment of the fine movements, as well as tingling in the fingers bilaterally. Neurophysiological examinations from four consecutive tests, performed in 2001, 2011, 2016 and 2018 are presented in Table 2. Nerve conduction studies (NCS) were consistent with severe demyelination and secondary axonal degeneration. There are absent sensory responses in both upper and lower limbs. At the age of 37 years the compound muscle action potentials (CMAPs) in the lower limbs were unobtainable. In the upper limbs, CMAPs had severely reduced CV, prolonged distal latencies and low amplitudes. Needle EMG showed scattered fibrillation potentials and neurogenic motor unit action potentials.

**Table 1:** Clinical comparison of all reported patients carrying *PMP2* mutations

Patient	A.III-1	A.II-2	B.II-2	B.II-1	Hong YB et al.			Motley W et al. 2016					Gonzaga-Jauregui et al. 2015	Punetha J et al. 2018
					II-4	III-1	III-3	Family I/II.3	Family I/III.2	Family II/II.3.1	Family II/IV.5	Family II/IV.1		
Mutation in *PMP2*	p.M114 T	p.M114 T	p.V115A	p.V115A	p.I43N	p.I43N	p.I43N	p.I52T	p.I52T	p.T51P	p.T51P	p.T51P	p.I43N	p.I52T
Sex	F	M	M	M	F	M	M	M	M	F	F	F	M	F
Age at onset	1.5 y	1 y	2–3 y	2–3 y	18 y	6 y	8 y	17 y	3 y	5 y	NR	18 mo	6	5
First symptoms	Delay in starting to walk, frequent falls	Walking with frequent falls	Slight clumsiness during physical activities	Slight clumsiness foot deformity	Distal leg muscle weakness	Frequent falling	Distal leg muscle weakness	Foot drop, frequent falling	Impaired coordination, frequent falling	Distal leg muscle weakness	Distal leg muscle weakness	Delay in starting to walk	NR	Distal leg muscle weakness
Age of involvement of upper limbs	29 y	34 y	40 y	40 y	41 y	20 y	28 y	NR	No involvement	NR	NR	No involvement	NR	NR
Age at last examination	37 y	34 y	44 y	45 y	58 y	38 y	36 y	48 y	13 y	37 y	13 y	24 y	NR	21
WEAKNESS: LOWER LIMBS (MRC SCALE)														
Knee flexion/extension	4/5	4/5	5/5	5/5	NR	NE	NE	5/5	5/5	NR	NR	5/5	NR	NR
Foot dorsiflexion	1/5	2/5	4/5	5/5	4/5	< 4/5	4/5	4−/5	3/5	0/5	NR	1/5	NR	NR
Plantar flexion	3/5	3/5	4/5	5/5	NR	NR	NR	5/5	5/5	0/5	NR	4/5	NR	NR
Great toe extension	0/5	1/5	4/5	5/5	NR	NR	NR	0/5	3/5	0/5	NR	0/5	NR	NR
WEAKNESS: UPPER LIMBS (MRC SCALE)														
Elbow flexion/extension	5/5	5/5	5/5	5/5	NR	NR	NR	5/5	5/5	NR	NR	5/5	NR	NR
Wrist flexion	4/5	5/5	5/5	5/5	NR	NR	NR	5/5	5/5	NR	NR	5/5	NR	NR
Wrist extension	4/5	5/5	5/5	5/5	NR	NR	NR	5/5	5/5	NR	NR	5/5	NR	NR
Finger flexion	3+/5	45	5/5	5/5	4/5	NR	< 4/5	5/5	5/5	NR	4/5	3–4/5	NR	NR
Finger extension	4/5	4/5	5/5	5/5	4/5	NR	< 4/5	5/5	5/5	NR	4/5	3–4/5	NR	NR
Thumb abduction	3/5	4/5	5/5	5/5	NR	NR	NR	4−/5	5/5	More affected than the other intrinsic hand muscles	4/5	1/5	NR	NR
REFLEXES														
Brachioradial	–	+/−	–	+/−	NR	NR	NR	NR	+/−	+/−	+	–	–	–
Biceps	+/−	+/−	–	–	NR	NR	NR	–	+/−	+/−	+	–	–	–
Triceps	+/−	+/−	–	–	NR	NR	NR	NR	+/−	+/−	+	–	–	–
Patellar	–	–	–	–	–	–	+/−	–	–	–	–	–	–	–
Achille	–	–	–	–	–	–	+/−	–	–	–	–	–	–	–
SENSATION MODALITIES: LOWER LIMBS														
Superficial	D	D	N	N	D	D	D	D	D	D	N	D	D	NR
Vibration	D	D	N	N	D	D	D	D	D	NR	N	D	D	NR
Romberg sign	+	+	NE	NE	NR	NR	NR	NR	NR	NR	N	+	NR	NR
SENSATION MODALITIES: UPPER LIMBS														
Superficial	N	N	N	N	D	D	D	N	N	NR	N	D	D	NR
Vibration	N	N	N	N	D	D	D	N	N	NR	N	D	D	NR
Independent ambulation	Preserved/steppage	Preserved/steppage	Preserved	Preserved	Preserved	Preserved	Preserved	Preserved	Preserved	Preserved with difficulties	Preserved	Preserved	NR	Preserved/steppage
Walking aids used	No	No	Orthopedic shoes	No	Ankle-foot orthoses	Ankle-foot orthoses	Ankle-foot orthoses	Ankle foot orthoses	NR	NR	NR	NR	NR	No
Skeletal deformities	Pes cavus and equinovarus	Pes cavus and equinovarus	Pes varus L > R	Pes varus	Pes cavus	Pes cavus	Pes cavus	NR	Pes cavus	Pes cavus and equinovarus	Pes equinovarus	Pes equinovarus	Pes cavus,	Pes cavus at the age of 12 y Pes planus at the age of 20 y
Foot deformity surgery	No	Yes, at the age of 26 y	On the left foot at the age of 41 y	No				NR	No	Achilles tendon lengthening Surgery at the age of 11	NR	At the age of 11 y	NR	Two on the right foot
Muscle atrophy	Severe for calf, peroneal, foot muscles and milder for thenar and hypothenar muscles	Moderate for calf, peroneal, foot muscles	Mild for calf, peroneal, foot muscles	No	Mild (U < L)	Moderate (U < L)	Mild (U < L)	NR	Atrophy of calf, peroneal, foot muscles	Atrophy of calf, peroneal, foot, intrinsic hand muscles	Atrophy of calf, peroneal, and foot muscles	Atrophy of calf, peroneal, and foot muscles	Atrophy of calf, peroneal, foot, intrinsic hand muscles	NR
CMT neuropathy score	15	NE	NE	NE	9	20	13	14	12	NR	NR	NR	NR	NR
9HPT	25.7 s	NE	NE	NE	22.7 s	31.4 s	26.3 s	NR	NR	NR	NR	NR	NR	NR
Any additional features	No	No	No	Epilepsy with generalized seizures since adolescence	Hand tremor	Hand tremor	Hand tremor	No	No	No	No	No	No	NR
Muscle MRI findings	Predominant signal changes in the anterior and lateral compartments of the lower leg muscles	NE	NE	NE	Predominant signal changes in the anterior and lateral compartments of the lower leg muscles	Predominant signal changes in the anterior and lateral compartments of the lower leg muscles	Predominant signal changes in the anterior and lateral compartments of the lower leg muscles	NE	NE	NE	NE	NE	NE	NE
Sural nerve biopsy	NE	NE	NE	NE	Onion bulbs and degenerating fibers with various myelin abnormalities	NE	NE	Onion bulbs, reduced density of myelinated axons	NE	NE	NE	NE	Demyelinating neuropathy; onion bulb formation	NE

*M*- male, *F*- female *mo*- months, *y*- years, *D*- decreased, *N*- normal, *L*-left, *R*- right, *9HPT*- nine hole peg test, *NE*- not examined, *NR*- not reported

**Table 2:** Electrophysiological recordings

	FAMILY A																FAMILY B							
	A.II-2				A.III-1 (proband)												B.II-1				B.II-2 (proband)			
Age at evaluation	34 years of age				20 years of age			30 years of age			35 years of age			37 years of age			46 years of age				37 years of age			
Sensory	CV (m/s)	A (μV)	DL (ms)		CV (m/s)	A (μV)	DL (ms)	CV (m/s)	A (μV)	DL (ms)	CV (m/s)	A (μV)	DL (ms)	CV (m/s)	A (μV)	DL (ms)	CV (m/s)	A (μV)	DL (ms)		CV (m/s)	A (μV)	DL (ms)	
Median	NR	NR	NR		NE	NE	NE	NR	NR	NR	NR	NR	NR	NR	NR	NR	54.9	5.9	NR		53.1	6.2	NR	
Ulnar	NR	NR	NR		NE	NE	NE	NR	NR	NR	NR	NR	NR	NR	NR	NR	53.1	2.4	NR		56.3	2.4	NR	
Sural	NR	NR	NR		NE	NE	NE	NR	NR	NR	NR	NR	NR	NR	NR	NR	43.3	6	NR		39.9	6.4	NR	
Motor	CV (m/s)	A (mV)	DL (ms)	FW-L (ms)	CV (m/s)	A (mV)	DL (ms)	CV (m/s)	A (mV)	DL (ms)	CV (m/s)	A (mV)	DL (ms)	CV (m/s)	A (mV)	DL (ms)	CV (m/s)	A (mV)	DL (ms)	FW-L (ms)	CV (m/s)	A (mV)	DL (ms)	FW-L (ms)
Median	12	3.1	5.13	NE	NE	NE	NE	8.9	8.3	6.4	8	4.1	6.13	6.5	4.3	5.6	49.3	16.5	3.7	33.8	39.6	14	3.5	47.7
Ulnar	14.7	2.9	5.8	NE	21.9	12.5	8.8	11.1	6.2	7.9	11.7	2.7	5.9	12.5	2.7	6.4	56.3	10.1	2.6	35.4	45.3	10.3	3.3	43
Peroneal	NR	NR	NR	NE	NR	4.3	14	NR	NR	NR	NR	NR	NR	NR	NR	NR	43.4	6.5	4.5	NE	30.6	0.7	4.1	NE
Tibial	NR	NR	NR	NE	NE	NE	NE	NR	NR	NR	NR	NR	NR	NR	NR	NR	47.5	9.5	4.5	60.3	35.9	4	4.2	76

*CV* – nerve conduction velocity, *DL* – distal latency, *FW-L* – F-wave measurements, *A* – amplitude, *NE*- not examined, *NR*- not recorded

The proband’s parent (A.II-2) started to walk at the age of 1 year with frequent falls. Foot deformities (high arched feet, thin calves) became apparent in the first decade of life. From the age of 34 years the patient reported weakness in the distal hand muscles with impairment of the fine movements. Neurological examination at the last follow-up (age 34) is presented in Table 1. NCS displayed a demyelinating pattern with secondary axonal degeneration in the lower limbs.

Lower limb musculature of patient A.III-1 was examined using magnetic resonance imaging (MRI) (Fig. 2b-e). The lateral compartments of the calves, including *peroneus longus* and *brevis* muscles were more severely affected, followed by *extensor digitorum brevis* and *tibialis anterior* muscles. The posterior compartment of the calves seemed relatively preserved. At thigh level the muscles were preserved as well, with slight changes visible in *semimembranosus* and *semitendinosus* muscles.

#### Family B

The proband (B.II-2) is a German individual who was adopted at the age of 1.5 years. The patient first exhibited a slight clumsiness with children’s gymnastics at the age of 2–3 years (Table 1). Foot deformities (*pes varus)* were observed at the age of 10 years, leading to walking impairment. After consultation with a child neurologist at youth, B.II-2 first presented in the neurological outpatient clinic at the age of 37 years. At that time, the patient complained of slightly progressive walking impairment and exercise induced muscle cramps in the upper limbs. The neurological examination was consistent with polyneuropathy with a predominant involvement of the lower limbs. The patient underwent surgical correction of foot deformity on the left side at the age of 41. When last examined at the age of 44, the motor and sensory deficits had not progressed significantly. B.II-2 was able to walk 2–3 km with orthopaedic shoes but without further aids. NCS performed at the age of 37 years showed a moderate reduction of motor and sensory amplitudes as well as conduction velocities in the lower limbs and markedly prolonged F-wave latencies, consistent with a primarily demyelinating pathology most pronounced on proximal parts of the motor nerve (Table 2). These remained without any major changes in the examination seven years later, at age 44.

The older sibling (B.II-1, also adopted) was reported to present with foot deformities and slight clumsiness since the age of 2–3 years. Since these complaints have not interfered with everyday activities, the individual was followed up at the age of 46 years. Patient B.II-1 revealed similar, albeit milder clinical features with *pes varus* deformity more pronounced on the right side, an inability to walk on heels, areflexia, but absence of muscle atrophy or sensory deficits (Table 1). As an additional clinical feature, B.II-1 had suffered from recurrent generalized seizures with an onset at the age of 12 years, which reduced markedly upon therapy with valproic acid. NCS revealed only discreet abnormalities with prolonged F-wave latencies in the lower and upper limbs, suggesting a focal demyelinating pathology at the level of the roots or the proximal parts of the motor nerves, but mostly normal motor and sensory amplitudes and conduction velocities. Interestingly, the CVs of the sensory nerves in the upper limbs were normal, while the amplitudes of SNAPs were decreased, suggesting axonal damage. The CVs of the median and ulnar nerves were decreased with normal amplitudes and distal latencies of CMAPs (Table 2).

Both adopted sibs originate from the same biological parents. One of them (B.I-2) was reported to have had a similar disease but no clinical data were available.

### Genetic analysis

We performed whole exome sequencing (WES) on the proband of the Bulgarian family (A.III-1) (Fig. 1a) as previous genetic analysis eliminated the *17p*-duplication involving *PMP22* and mutations in 29 common CMT-causing genes. A coverage of 10x over 85% of the sequence was reached using the SeqCap EZ Exome Kit v2 (Roche, Basel, Switzerland) on an Illumina NextSeq500 analyser (Illumina, San Diego, CA). A total of 21,870 sequence variants in protein-coding regions were identified exome-wide. The variant filtering was performed using a dominant disease model and a frequency cut-off of < 1% in gnomAD and ExAC online genomic databases [19]. Variants from 329 genes associated with CMT, SMA, HMN, SCA, HSP and ALS were extracted, leading to the identification of a novel variant in *PMP2* p.Met114Thr (c.341C > T) that co-segregated with the disease phenotype in family A (Fig. 1a, b). No other candidate variants were identified in the known disease-causing genes.

Following this finding, we performed a genetic screening of *PMP2* in 241 patients with a clinical diagnosis of CMT1 or I-CMT, an unknown genetic cause and mutations in the most common CMT1 genes (*PMP22*, *MPZ* and *GJB1*) previously excluded (Table S1). We identified another novel missense mutation in *PMP2* p.Val115Ala (c.344 T > C) in a German patient (B.II-2) that co-segregated with the disease in the two affected brothers (Fig. 1a, b). Their biological parents were not available for segregation analysis. To eliminate the presence of other disease-causing mutations in known CMT-causing genes, whole exome sequencing was performed on the proband (B.II-2) using the same procedure as described above and reaching 96% at 10x coverage. In the resulting 23,844 variants within protein-coding regions we did not identify any other candidates in known CMT-causing genes. Considering the electrophysiological findings in the German patients, we subsequently expanded our mutation screening to a cohort of 352 patients diagnosed with CMT2, but no additional *PMP2* variants were identified.

The newly identified mutations (p.Met114Thr; p.Val115Ala) are not reported in gnomAD. They both target conserved nucleotide (GERP = 5.89; 4.71) and amino acid residues that, interestingly, are adjacent to each other (Fig. 1c, e). These residues are surrounded by fatty acid coordinating residues (Fig. 1d, f) [15]. Both changes were predicted to be disease-causing by the available online mutation prediction algorithms.

The crystal structure of the wildtype PMP2 protein was used to estimate how the mutations could impact protein stability. The “ΔΔG” measurement (Yasara v17.12.24) provides a comparison of the predicted change in free energy between mutated and wildtype proteins [20, 21]. Positive values indicate an increase in free energy suggesting a destabilization, while negative values point to a stabilizing effect. Both PMP2 p.Met114Thr and p.Val115Ala variants are predicted to have a destabilizing effect on the overall protein structure with a ΔΔG > 2, which is comparable to the previously reported PMP2 mutations (Fig. 1e).

## Discussion

Overall, the clinical onset and the rate of progression of the reported PMP2 patients demonstrate broad variability with initial complaints between the first years of life with delayed motor milestones to adolescence [11–13]. Foot deformities and peroneal weakness are invariably present in all affected individuals [11–13]. A comparison between all patients with known *PMP2* mutations is presented in Table 1.

Although the two novel mutations are in close structural proximity, families A and B demonstrate significant variability of disease severity in terms of clinical features and electrophysiologic pattern of involvement. In family A, the onset was very early with distal weakness in lower limbs in the first year of life and delayed walking in patient A.III-1, whereas in family B both affected individuals exhibited only a slight clumsiness and *pes varus* in the first decade of life, that did not interfere much in their subsequent functioning. Regardless of the age at onset the course of the polyneuropathy in both families was quite benign, with preserved ambulation until the last follow-up performed in the 4th–5th decade. Hand muscle weakness appeared in the third-forth decades in the Bulgarian family, while the German patients showed no such signs until their latest follow-up (5th decade). In line with previous reports, the Bulgarian patients exhibited a predominant peroneal involvement in the lower limbs and a more severe weakness of *m. abd. pollicis* in comparison to other hand muscles (Table 1) [11–14].

Nerve conduction studies of the PMP2 patients reported up to now demonstrate classical features of demyelinating polyneuropathy with very slow CVs of the motor and sensory nerves below 20 m/s and secondary axonal degeneration [12–14]. NCS in all examined affected individuals presented in this report were consistent with a demyelination of variable severity and location. Electrophysiological recordings in the Bulgarian family revealed very slow CVs of the motor fibres of median and ulnar nerves, not exceeding 15 m/s (Table 2), while CVs of the motor nerves in the lower limbs and CVs of the sensory nerves of both upper and lower limbs were unobtainable, as previously reported [12, 13]. In contrast, NCS performed in the fifth decade in the German patients displayed very mild changes, consistent with a primarily demyelinating focal pathology most pronounced at the proximal parts of the motor nerves and sensory axonal degeneration in the upper limbs in one of the patients, which is in line with their milder clinical features. Because of this unusual NCS pattern, patients in family B do not fulfill the classical criteria neither for demyelinating neuropathy (except for the prolonged F-wave latencies), nor for axonal neuropathy. Although the reason for this peculiar NCS features remain unknown, our findings suggest that *PMP2* testing should come into consideration in patients with even subtle electrophysiological signs of demyelination.

The muscle MRI in patient A.III-1 showed a more severe fatty replacement in the calf muscle compared to the thigh muscle, which is consistent with a length-dependent degeneration of the axons. As observed previously, the peroneal muscles were more severely affected compared to tibialis anterior muscles [12]. The posterior lower leg compartment was less involved, which is similar to what is observed in CMT1A patients.

The three previously reported PMP2 mutations (p.Ile43Asn, p.Thr51Pro, p.Ile52Thr) cluster in two neighbouring beta-strands β2-β3 (Fig. 1e). Crystallography studies show that all of them induce an increased aggregation tendency and a significant destabilization of the tertiary structure of the protein, with p.Thr51Pro showing the strongest effect [16]. The two new mutations (p.Met114Thr and p.Val115Ala) form a second mutational cluster on β9 (Fig. 1e). Like the known mutations, they affect conserved residues amongst the PMP2 orthologs and throughout the FABP family (Fig. 1c, d). The introduction of residues with different polarity (p.Met114Thr) or flexibility (p.Val115Ala) could disturb the protein structure in a similar manner as demonstrated with previously reported mutations, however further studies are required to confirm this hypothesis [16, 22]. PMP2 expression is restricted to Schwann cells, as reported in mice and confirmed by our immunoblotting experiments (data not shown) [18]. Unfortunately, we did not detect ectopic PMP2 expression in EBV-transformed lymphocytes from patients and controls. Therefore, we could not investigate the validity of the in silico predictions and whether the two missense mutations cause protein destabilisation or possible aggregation in vivo*.*

Concerning its fatty acid binding properties, PMP2 was shown to have affinity towards key components of the myelin sheath including palmitate, oleate and cholesterol [16, 23]. Importantly, the three known CMT1-causing mutations alter the conformational dynamics, leading to differential fatty acid binding properties when compared to wildtype PMP2 [16]. The new mutations lie in a protein region structurally surrounded by fatty acid-coordinating residues (p.Arg107, p.Arg127, p.Tyr129) (Fig. 1f), suggesting their potential detrimental effect on ligand binding.

The mechanism through which *PMP2* mutations cause CMT is currently unknown. This is partly due to the lack of complete understanding of the function of PMP2. A Pmp2 knock-out (*Pmp2*^*KO*^) mouse model showed that the protein is not required for the formation of the myelin sheath [18]. The *Pmp2*^*KO*^ mice largely resemble their wildtype littermates, unlike what is observed in corresponding studies with *Mpz* or *Pmp22* null-mice [18, 24, 25]. However, the myelin was found to be thinner than in wildtype mice and did not recover as well under nerve crush injury, suggesting possible functions for Pmp2 in the myelin repair [18]. Together with the findings from crystallography studies these data suggest that the complete loss of function is not the disease driving mechanism, likely the mutations induce a gain of toxic function resulting in an unstable myelin sheath [16].

## Conclusion

Here we report two mutations in *PMP2* as novel causes of dominant CMT that is primarily demyelinating in nature. The new mutations affect adjacent amino acid residues, revealing a novel mutation cluster in PMP2. Importantly, we expand the clinical and electrophysiological spectrum of *PMP2*-related neuropathy with the identification of very mildly affected individuals who have only subtle proximal demyelination and focal pattern of distribution along peripheral nerves. Our findings contribute to an improved clinical and genetic diagnosis of patients and families with inherited peripheral neuropathies.

## Methods

### Clinical and electrophysiological evaluation

The patients were interviewed to obtain information on family history, age at onset, initial symptoms, distribution of muscle weakness, disease progression, and current disability. The four mutation carriers were subjected to neurological examination including testing of muscle strength according to the Medical Research Council (MRC) grading method. Serial clinical and electrophysiological evaluation spanning 17 years was performed on one of the patients (A.III-1).

### Magnetic resonance imaging

Lower limb musculature of patient A.III-1 was examined using a 3 T MRI scanner (Siemens Magnetom Verio 3.0 T, Tarrytown, USA). Non-enhanced T1, T2 and proton density fat saturated sequences were applied in pelvis, bilateral thighs and lower legs.

### Patient cohorts

The initial cohort consisted of isolated patients with a clinical diagnosis of demyelinating (*n* = 174) or intermediate (*n* = 67) peripheral neuropathy. Based on the clinical findings in family B we expanded the *PMP2* screening to 352 additional CMT2 index patients. The most common causes of CMT, including *17p* duplication and mutations in *PMP22, MPZ, MFN2* and *GJB1* were excluded prior to the onset of this study*.* The full list of genes excluded prior to whole exome sequencing (WES) analysis in patients A.III-1 and B.II-2 is provided in Additional file 1: Table S1.

### Whole exome sequencing

WES of individuals A.III-1 and B.II-2 were performed at the Neuromics Support Facility of the VIB Centre for Molecular Neurology, Antwerp, Belgium. The sequencing was done using SeqCap EZ Exome Kit v3 (Roche, Basel, Switzerland) on an Illumina NextSeq500 system (Illumina, San Diego, CA, USA) to an average of 10-fold coverage over 85% and 96% of the targeted regions for A.III-1 and B.II-2 respectively. The paired-end reads were then aligned to the human reference genome GRCh37/hg19 using Burrows-Wheeler aligner (0.7.15-r1140) [26]. In both patients the variant calling was performed using the genome analysis toolkit (GATK) versions 3.7 (A.III-1) and 4.0.6.0 (B.II-2) [27]. Additional individual analyses were done using the sequence alignment/map tools (SAMtools, v1.6) for A.III-1 and Strelka (v2.9.9) for B.II-2 [28, 29]. GenomeComb software was used to extract the sequencing gaps in known CMT1-causing genes and to perform variant annotation and filtering [30]. In both individuals sequence gaps were closed in the CMT1 causing genes using Sanger sequencing.

### *PMP2* sequencing

All four exons of *PMP2* (RefSeq NM_002677.4) were amplified using the primers provided in Additional file 1: Table S2. The PCR products were subsequently purified with ExoSAP-IT (Affymetrix, Santa Clara, CA). We performed Sanger sequencing using the BigDye v3.1 Terminator cycle sequencing kit (Thermo Fischer Scientific, Waltham, MA). The fragments were electrophoretically separated on a 3730xl DNA Analyzer (Applied Biosystems, Foster City, CA, USA) with the alignment and analysis performed using SeqMan v5.07 (DNAStar, Madison, WI, USA). The nucleotide and amino acid numbering used in this study are according to the *PMP2* mRNA (NM_002677.4) and protein (CAG46538.1) sequences available at the National Centre for Biotechnology Information, and mutation description was according to Human Genome Variation Society nomenclature (http://varnomen.hgvs.org/).

### Prediction of mutation pathogenicity

To predict the possible impact of the mutation we used a combination of PolyPhen2 (v2.2.2), MutationTaster (v2013), SIFT (v6.2.0) and CADD (v1.3) [31–34]. The scores given by these programs are provided in Additional file 1: Table S3.

### Mutation modelling

The modelling of wild-type and mutant proteins was performed using Yasara v17.12.24 and Chimera v1.12 on the PMP2 (PDB ID: 3NR3) crystal structure [20, 35]. The effect of the amino acid change to the protein structure was estimated with FoldX v3.0b4 using standard parameters [21].

## Additional file

Additional file 1: **Table S1.** Genomic defects and genes analyzed prior to WES. **Table S2.** Primers used for PMP2 screening. **Table S3.** Scores from the pathogenicity prediction programs. (PDF 62 kb)

## Data Availability

All data generated or analysed during this study are included in this published article and its additional files.

## Abbreviations

ALS: Amyotrophic lateral sclerosis
CMAP: Compound muscle action potential
CMT: Charcot-Marie-Tooth
EBV: Ebstein-Barr virus
EMG: Electromyography
ExAC: Exome Aggregation Consortium
GJB1: Gap junction beta-1
gnomAD: Genome Aggregation Database
HMN: Hereditary motor neuropathy
HSP: Hereditary spastic paraplegia
MBP: Myelin basic protein
MFN2: Mitofusin 2
MPZ: Myelin protein zero
MRC: Medical Research Council
MRI: Magnetic resonance imaging
NCS: Nerve conduction studies
NCV: Nerve conduction velocities
NGS: Next generation sequencing
PDB: Protein data bank
PMP2: Peripheral myelin protein 2
PMP22: Peripheral myelin protein 22
SCA: Spinocerebellar ataxia
SMA: Spinal muscular atrophy
WES: Whole exome sequencing
GERP: Genomic evolutionary rate profiling
SIFT: Sorting tolerant from intolerant
CADD: Combined annotation dependent depletion

## References

[1] Barreto LC, Oliveira FS, Nunes PS (2016). Epidemiologic study of Charcot-Marie-tooth disease: a systematic review. Neuroepidemiology..

[2] Pareyson D, Saveri P, Pisciotta C (2017). New developments in Charcot-Marie-tooth neuropathy and related diseases. Curr Opin Neurol.

[3] Dyck PJ, Lambert EH (1968). Lower motor and primary sensory neuron diseases with peroneal muscular atrophy. Arch Neurol.

[4] Thomas PK, Calne DB (1974). Motor nerve conduction velocity in peroneal muscular atrophy: evidence for genetic heterogeneity. J Neurol Neurosurg Psychiatry.

[5] Bradley WG, Madrid R, Davis CJ (1977). The peroneal muscular atrophy syndrome. Clinical genetic, electrophysiological and nerve biopsy studies. Part 3. Clinical, electrophysiological and pathological correlations. J Neurol Sci.

[6] Schmitt S, Castelvetri LC, Simons M (1851). Metabolism and functions of lipids in myelin. Biochim Biophys Acta.

[7] Greenfield S, Brostoff S, Eylar EH, Morell P (1973). Protein composition of myelin of the peripheral nervous system. J Neurochem.

[8] Lupski JR, de Oca-Luna RM, Slaugenhaupt S (1991). DNA duplication associated with Charcot-Marie-tooth disease type 1A. Cell..

[9] Timmerman V, Nelis E, Van Hul W (1992). The peripheral myelin protein gene PMP-22 is contained within the Charcot-Marie-tooth disease type 1A duplication. Nat Genet.

[10] Rossor AM, Polke JM, Houlden H, Reilly MM (2013). Clinical implications of genetic advances in Charcot–Marie–tooth disease. Nat Rev Neurol.

[11] Gonzaga-Jauregui C, Harel T, Gambin T (2015). Exome sequence analysis suggests that genetic burden contributes to phenotypic variability and complex neuropathy. Cell Rep.

[12] Hong YB, Joo J, Hyun YS (2016). A mutation in PMP2 causes dominant demyelinating Charcot-Marie-tooth neuropathy. PLoS Genet.

[13] Motley WW, Palaima P, Yum SW (2016). De novo PMP2 mutations in families with type 1 Charcot–Marie–tooth disease. Brain..

[14] Punetha J, Mackay-Loder L, Harel T (2018). Identification of a pathogenic PMP2 variant in a multi-generational family with CMT type 1: clinical gene panels versus genome-wide approaches to molecular diagnosis. Mol Genet Metab.

[15] Ruskamo S, Yadav RP, Sharma S (2014). Atomic resolution view into the structure-function relationships of the human myelin peripheral membrane protein P2. Acta Crystallogr D Biol Crystallogr.

[16] Ruskamo S, Nieminen T, Kristiansen CK (2017). Molecular mechanisms of Charcot-Marie-tooth neuropathy linked to mutations in human myelin protein P2. Sci Rep.

[17] Stettner M, Zenker J, Klingler F (2018). The role of peripheral myelin protein 2 in Remyelination. Cell Mol Neurobiol.

[18] Zenker J, Stettner M, Ruskamo S (2014). A role of peripheral myelin protein 2 in lipid homeostasis of myelinating Schwann cells. Glia..

[19] Karczewski KJ, Francioli LC, Tiao G (2019). Variation across 141,456 human exomes and genomes reveals the spectrum of loss-of-function intolerance across human protein-coding genes: supplementary information.

[20] Krieger E, Vriend G (2015). New ways to boost molecular dynamics simulations. J Comput Chem.

[21] Schymkowitz J, Borg J, Stricher F, Nys R, Rousseau F, Serrano L (2005). The FoldX web server: an online force field. Nucleic Acids Res.

[22] Huang F, Nau WM (2003). A conformational flexibility Scale for amino acids in peptides. Angew Chem.

[23] Majava V, Polverini E, Mazzini A (2010). Structural and functional characterization of human peripheral nervous system myelin protein P2. PLoS One.

[24] Giese KP, Martini R, Lemke G, Soriano P, Schachner M (1992). Mouse P0 gene disruption leads to hypomyelination, abnormal expression of recognition molecules, and degeneration of myelin and axons. Cell..

[25] Saporta MA, Katona I, Zhang X, et al. Neuropathy in a human without the PMP22 gene. Arch Neurol. 2011;68. 10.1001/archneurol.2011.110 PMC3711535 21670407

[26] Li H, Durbin R (2010). Fast and accurate long-read alignment with burrows–wheeler transform. Bioinformatics..

[27] McKenna A, Hanna M, Banks E (2010). The genome analysis toolkit: a MapReduce framework for analyzing next-generation DNA sequencing data. Genome Res.

[28] Li H (2011). A statistical framework for SNP calling, mutation discovery, association mapping and population genetical parameter estimation from sequencing data. Bioinformatics..

[29] Saunders CT, Wong WSW, Swamy S, Becq J, Murray LJ, Cheetham RK (2012). Strelka: accurate somatic small-variant calling from sequenced tumor–normal sample pairs. Bioinformatics..

[30] Reumers J, De Rijk P, Zhao H (2012). Optimized filtering reduces the error rate in detecting genomic variants by short-read sequencing. Nat Biotechnol.

[31] Adzhubei IA, Schmidt S, Peshkin L (2010). A method and server for predicting damaging missense mutations. Nat Methods.

[32] Vaser R, Adusumalli S, Leng SN, Sikic M, Ng PC (2016). SIFT missense predictions for genomes. Nat Protoc.

[33] Schwarz JM, Cooper DN, Schuelke M, Seelow D (2014). MutationTaster2: mutation prediction for the deep-sequencing age[letter]. Nat Methods.

[34] Rentzsch P, Witten D, Cooper GM, Shendure J, Kircher M (2019). CADD: predicting the deleteriousness of variants throughout the human genome. Nucleic Acids Res.

[35] Pettersen EF, Goddard TD, Huang CC (2004). UCSF chimera--a visualization system for exploratory research and analysis. J Comput Chem.

