# Supplementary material for: Peripheral myelin protein 2 – a novel cluster of mutations causing Charcot-Marie-Tooth neuropathy
Source: Orphanet J Rare Dis. 2019 Aug 14;14:197. doi: 10.1186/s13023-019-1162-x (PMC6692960; doi:10.1186/s13023-019-1162-x)
Supplement: Supplementary file 1 — Table S1. Genomic defects and genes analyzed prior to WES. Table S2. Primers used for PMP2 screening. Table S3. Scores from the pathogenicity prediction programs. (PDF 62 kb) [file 13023_2019_1162_MOESM1_ESM.pdf]

Palaima et al.

Additional information

Table S1. Genomic defects and genes analyzed prior to WES.

Gene	A.III-1	B.II-2
17p dupl.	+	+
PMP22	+	+
MPZ	+	+
GJB1	+	+
NDRG1	+	+
YARS	+	
GARS	+	
AARS	+	
MFN2	+	
GDAP1	+	+
SH3TC2	+	
HINT1	+	
BSCL2	+	
CTDP1	+	
DNM2	+	
FGD4	+	
GAN	+	
HSP22 (HSPB8)	+	
HSP27 (HSPB1)	+	
IGHMBP2	+	
GNB4	+	
LITAF	+	
MTMR2	+	+
NEFL	+	
NTRK1	+	
PRX	+	
RAB7	+	
SPTLC1	+	
SPTLC2	+	
TRPV4	+	

"**+**" - screening of the gene performed.

Table S2. Primers used for *PMP2* screening.

Exon	Forward primer	Reverse primer
**1**	5'-AGCCTTGCAAAACTCCCCAG-3'	5'-TTGCTTAGTCCCGACACTGC-3'
**2**	5'-TGTCTCAGGAGGTACACTCC-3'	5'-CCACTGAGCGTAGGATGTGG-3'
**3**	5'-AAAATCTTGGGTCAGTACTTGA-3'	5'-TGGTTCCATACTGAAAGAACTGC-3'
**4**	5'-ACTGGAGACTGACATACTGAT-3'	5'-CTCCTCTGGCCCCTGTCA-3'

Table S3. Scores from the pathogenicity prediction programs.

cDNA	Protein	SIFT (max score 0)	PolyPhen2 (max score 1)	Mutation Taster (max score 1)	CADD (>20)
c.341T>C	p.Met114Thr	0.01 (Del)	1 (PD)	1 (DC)	27.6
c.344T>C	p.Val115Ala	0.02 (Del)	0.923 (PD)	1 (DC)	25.3

Abbreviations: "Del" – deleterious, "DC" – disease causing, "PD" – probably damaging. A CADD score >20 suggests that the variant is amongst the top 1% of deleterious variants in the human genome.
